# Sustainable Dielectric Films with Ultralow Permittivity from Soluble Fluorinated Polyimide

**DOI:** 10.3390/molecules28073095

**Published:** 2023-03-30

**Authors:** Hejian Li, Xiangyi Kong, Shixiao Wang, Min Gong, Xiang Lin, Liang Zhang, Dongrui Wang

**Affiliations:** 1 Department of Chemistry and Chemical Engineering, School of Chemistry and Biological Engineering, University of Science and Technology Beijing, Beijing 100083, China; 2 Beijing Key Laboratory for Bioengineering and Sensing Technology, University of Science and Technology Beijing, Beijing 100083, China

**Keywords:** low dielectric constant, soluble polyimide, nonsolvent-induced phase separation, porous structure, recyclability

## Abstract

In the rapidly growing area of high-frequency communications, polyimide films with ultralow dielectric constant and dielectric loss, adequate insulating strength, and recyclability are in high demand. Using a synthesized soluble fluorinated polyimide, a series of recyclable porous dielectric films with varying porosities were fabricated in this study through nonsolvent-induced phase separation. By manipulating the mass ratio of the binary solvent used to dissolve the polyimide, the shape, size, and size distribution of the pores generated throughout the polyimide matrix can be accurately regulated. The porosity and average pore size of the as-prepared porous films were adjustable between 71% and 33% and between 9.31 and 1.00 μm, respectively, which resulted in a variable dielectric constant of 1.51–2.42 (100 kHz) and electrical breakdown strength of 30.3–119.7 kV/mm. The porous sPI film with a porosity rate of 48% displayed a low dielectric constant of 2.48 at 10 GHz. Coupled with their superior thermal stability, mechanical characteristics, and recyclability, these porous polyimide films are highly promising for constructing high-frequency microelectronic devices.

## 1. Introduction

With the rapid progress of high-frequency microwave communication technologies, an urgent need for high-performance low-dielectric materials has risen. These dielectric materials with low dielectric constant can decrease the capacitance between metal interconnects, resistance–capacitance delay, line-to-line crosstalk noise, and power dissipation, which makes them suitable as high-frequency, low-loss boards; semiconductor packaging materials (e.g., chip modules); and interlayer dielectrics (e.g., transistor devices) [1,2,3,4,5,6,7,8]. For example, dielectrics in printed circuit boards must have a dielectric constant (*k*) value of <2.5 and a dissipation factor of <0.002 at frequencies greater than 5 GHz in order to meet the criteria of 5G mobile communication in terms of reliability and signal delay [9,10,11]. A variety of polymers, including polyimide (PI), polytetrafluoroethylene (PTFE), polystyrene (PS), polypropylene (PP), and polycarbonate (PC), have been extensively investigated as promising candidates for low-*k* materials. However, conventional polymers such as PS and PP have limited maximum operating temperatures and relatively poor mechanical properties, which cannot meet the increasing requirements in diverse application scenarios [12,13]. Aromatic polyimides have been widely employed as dielectric materials for electronic packaging and electrical insulation in the microelectronics industry due to their superior thermal, mechanical, and dielectric properties [14,15,16,17,18]. Nevertheless, the *k*-value of conventional polyimides, such as commercial DuPont Kapton polyimide films, is typically between 3.1 and 3.5, which is insufficient for high-frequency microelectronics. In addition, aromatic polyimides with hard aromatic backbones have been traditionally synthesized via a two-step polymerization: (1) condensation polymerization between dianhydrides and diamines under mild conditions in a strong polar aprotic solvent (e.g., N,N-dimethylacetamide (DMAc), N-methylpyrrolidone (NMP), and dimethylsulfoxide (DMSO) to obtain the corresponding polyamide (PAA); and (2) thermal imidization at around 300 °C to convert PAAs into thermosetting PIs. Apparently, the high temperature needed for imidization may raise the risk of thermal damage to microelectronic components during manufacturing [19,20]. In the meantime, the thermosetting characteristic of polyimides, despite providing great dimensional stability and thermal stability, renders electronic devices unrecyclable and destined to generate a substantial amount of electronic trash. Therefore, over the past few years, there has been considerable interest in the development of soluble polyimide (sPI) with low *k*, low dielectric loss, high mechanical strength, easy recycling properties, as well as other good overall performance.

It is well known that the dielectric properties of materials are related to the degree of polarization. There are four types of polarization (interface polarization, orientation polarization, electronic polarization, and atomic polarization) in dielectric materials, and the response is strongly dependent on the frequency of the electric field. To create low-dielectric materials, one can reduce either the dipole strength or the number of dipoles, or use a combination of the two [21,22,23,24,25,26,27,28,29,30,31,32,33,34,35]. Decreased dipole polarization, molecular order, and intermolecular interactions can be achieved simultaneously by introducing a variety of weakly polar chemical groups into the chain structure in order to realize low-*k* sPI [23]. For instance, Hu and coworkers synthesized a type of sPI containing large volumes of fluorene- and pyridine-containing units [23]. The sPI was soluble in common organic solvents and possessed a low dielectric constant (2.22–3.09 at 10 MHz). Zheng et al. synthesized a series of sPIs using three distinct commercial aromatic dianhydrides and an aromatic diamine with an asymmetric pendant terphenyl group [34]. The resulting sPI films exhibited relatively low dielectric constants (2.64). However, the high cost of monomers and the complexity of the synthesis limited the practical applications of such low-*k* sPI materials. Introducing porous structure into the sPI matrix, which effectively decreases the packing density of dipoles, is another method for achieving low-*k* sPI. Consequently, the incorporation of air, which has an extremely low dielectric constant of nearly 1.0, can significantly reduce the dielectric constant of resulting porous sPI. However, thus far, relatively few studies have been conducted along this path to develop low-*k* sPI films [36,37,38]. As a comparison, a significant amount of research has focused on the fabrication of conventional thermosetting polyimides with low *k* by introducing porosity [39,40,41,42]. In this case, the formation of porous structures is generally realized in two steps. First, pores can be incorporated into soluble precursor PAAs by directly creating air pores [39] or by introducing porous fillers such as mesopore silica nanoparticles [40,41]. The porous PAA films are then converted to porous polyimides at elevated temperatures via thermal imidization. As mentioned above, the preparation of thermosetting polyimides using the two-step technique is harsh, and the resulting dielectrics are not recyclable.

Herein, we present a simple method for creating low-*k* sPI films with controllable porosity architectures and low-dielectric characteristics. We built porous structures across the films using a fluorinated sPI as the starting material and utilizing a nonsolvent-induced phase separation technique at a low temperature of 0 °C. By adjusting the mass ratio of the binary solvent (N,N-dimethylformamide and chloroform), the resulting sPI films’ pore size and porosity can be precisely controlled. The relationship between the developed sPI films’ porosity and their mechanical and dielectric properties was also thoroughly studied. In contrast to traditional porous polyimide films, the low-*k* sPI films presented could be continuously recycled and remanufactured into low-*k* dielectric materials without compromising their performance.

## 2. Results and Discussion

**Figure 1:**
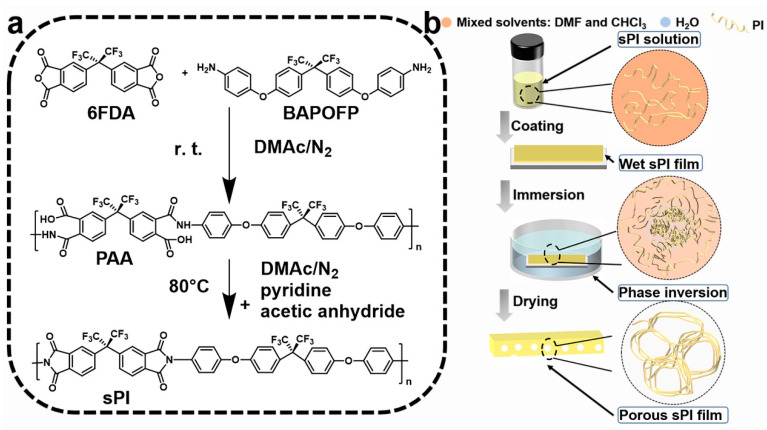
(**a**) Synthetic route for the soluble polyimide; (**b**) schematic illustration of a nonsolvent-induced phase separation approach for the fabrication of porous films.

**Figure 2:**
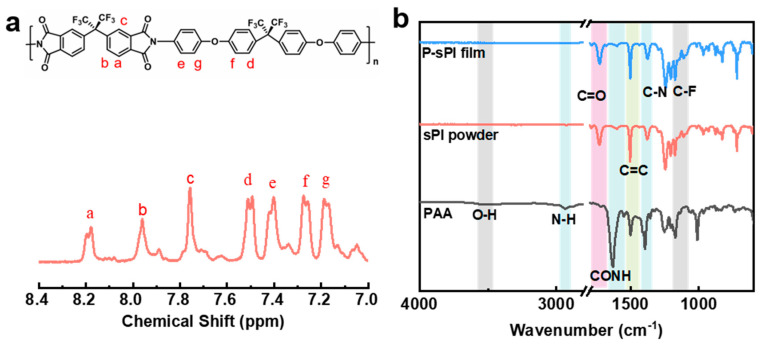
(**a**) ^1^H-NMR spectrum of sPI by using d^6^-DMSO as the solvent; (**b**) FTIR spectra of intermediate PAA, synthesized sPI, and porous sPI film.

As shown in Figure 1, one type of soluble fluorinated polyimide was synthesized and fabricated into porous low-*k* films via a nonsolvent-induced phase separation technique (also known as delayed phase inversion). The manufacturing procedures for porous films included three steps: (1) sPI solutions in mixed DMF/CHCl_3_ were blade-coated onto pre-cleaned glass plates with predetermined thicknesses; (2) wet sPI films were transferred into a water bath to form the porous structures through a solvent exchange process; and (3) porous sPI films were dried in vacuum and peeled off from the glass plates. The sPI was synthesized using a one-pot method with a fluorinated dianhydride (6FDA) and a fluorinated diamine (BAPOFP) as the monomers [43,44]. The reaction was initially conducted at room temperature to produce PAAs with desired viscosity. Then, acetic anhydride and pyridine were introduced, and the temperature was increased to 80 °C to accelerate the imidization. The chemical structure of the resultant sPI was characterized using ^1^H-NMR and FTIR. As shown in Figure 2a, all the protons located in the aromatic rings on the sPI film’s backbones exhibited corresponding peaks in the range of 7.1–8.2 ppm: δ (ppm): 8.18 (d, Ar-H); 7.95 (d, Ar-H); 7.74 (s, Ar-H); 7.50 (d, Ar-H); 7.40 (d, Ar-H); 7.26 (d, ArH); and 7.17 (d, Ar-H). The FTIR spectra of PAA, sPI, and porous sPI film are compared in Figure 2b. The emergence of sPI peaks at 1782, 1730, and 1380 cm^−1^, which are attributed to the C=O asymmetrical stretching, C=O symmetrical stretching, and C–N stretching, respectively, indicates the existence of imide rings, while the complete removal of PAA peaks at 3500 cm^−1^ (O–H) and 1660 cm^−1^ (C=O) suggests a high degree of imidization. The bands in the range of 1300–1100 cm^−1^ corresponding to C–F stretching reveal that fluorine atoms were successfully incorporated into the sPI backbones. In addition, the nearly identical IR spectra of synthesized sPI and porous sPI film suggest that the phase separation process had little influence on the chemical structure of the polymer. According to GPC analysis, the resultant sPI had an *M*_n_ of 89,000 and a PDI of 2.53 (using DMF as the elution agent and PS as standards). All of these results confirm that a soluble fluorinated polyimide with a relatively high polymerization degree was achieved.

**Figure 3:**
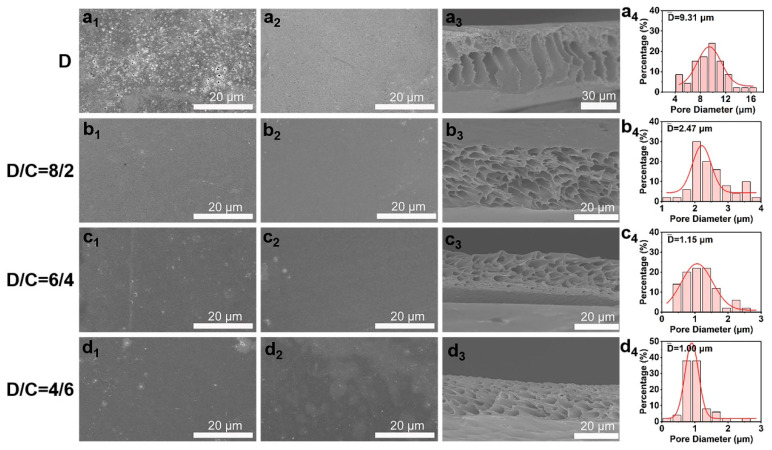
Typical SEM images of top surfaces (**a_1_**–**d_1_**), bottom surfaces (**a_2_**–**d_2_**), and cross-sections (**a_3_**–**d_3_**) of porous sPI films using mixed DMF/CHCl_3_ (D/C) solvents with various weight ratios. Panels (**a_4_**–**d_4_**_)_ displaying pore size distributions in the porous sPI films.

The synthesized sPI powders were then used in the nonsolvent-induced phase separation procedure to create porous films. To produce pores throughout the sPI matrix, we selected mixtures of DMF and CHCl_3_ as solvents and water as the nonsolvent. CHCl_3_ is not miscible with water, in contrast to DMF. The addition of a suitable amount of CHCl_3_ affects the properties of the stock solution and inhibits the formation of macrovoids. Consequently, the pore size and distribution in the resulting sPI films can be precisely controlled by adjusting the weight ratio of DMF/CHCl_3_ (D/C) in the starting solution. Figure 3 depicts the surface and cross-sectional morphologies of the resultant sPI films. When pure DMF was used as the solvent, finger-shaped cavities were formed within the sPI film, along with large holes on its top and bottom surfaces. The top and bottom surfaces of the resulting films grew denser when CHCl_3_ was added to the mixture of solvents used to dissolve sPI with DMF. In the meantime, the created voids within the films evolved into cavernous pores. As shown in Table 1, the porosity and average pore diameter steadily decreased from 71% to 33% and from 9.37 to 1.00 μm, respectively, as the CHCl_3_ content rose from 0 wt% to 60 wt%. In order to understand the influence of the D/C ratio on the porosity formation, we calculated the solubility parameters of each component involved in the phase separation process, and the results are shown in Table 2 (the calculation details are listed in Supplementary Materials). The difference in solubility parameter between the mixed solvents and nonsolvent (Δδ_mix−water_) increased with the increase in CHCl_3_ content, which is ascribed to the weak affinity of CHCl_3_ to water. A larger Δδ_mix−water_ indicates a slower exchange rate between the mixed solvents and nonsolvent when immersing wet sPI films into the water bath. Consequently, sPI chains had more time to adjust their conformation during the phase separation process, resulting in a porous structure with smaller pores and narrower size distribution.

**Figure 4:**
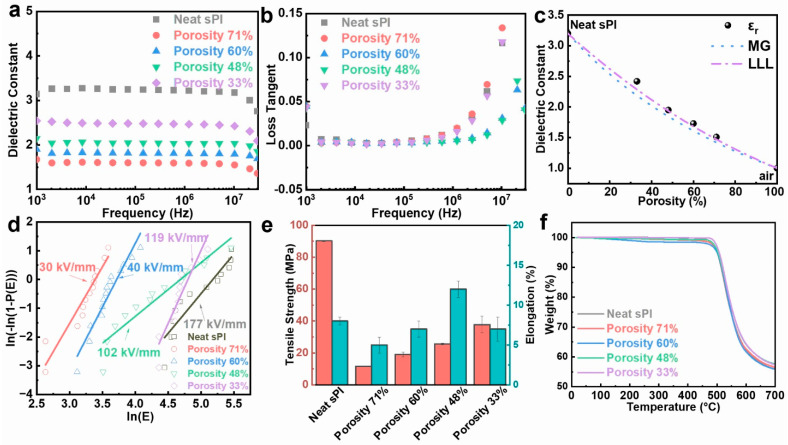
Performance of porous polyimide films with different porosities: (**a**) dielectric constant and (**b**) loss tangent; (**c**) dielectric constant at 10^5^ Hz vs. porosity of the porous sPI films. The measured data are compared with predictions from two models of composite: Maxwell–Garnett (dotted line), Looyenga–Landau–Lifshitz (dotted–dashed line); (**d**) electric breakdown strength; (**e**) tensile strength and elongation at break; (**f**) thermal stability.

Owing to the incorporation of microvoids, which had the lowest relative dielectric constant of 1.0, the porous sPI films showed significantly low-*k* values. Variations in the dielectric constant and dielectric loss tangent of porous sPI films as a function of frequency in the range of 10^3^ to 10^7^ Hz at room temperature are shown in Figure 4a,b. Taking the *k* values at 10^5^ Hz as examples, the porous sPI films revealed low-*k* values of 1.51–2.42, which were much lower than that of dense sPI films (3.20). As depicted in Figure 4c, the *k* values of sPI films were inversely proportional to their porosity. The sPI film with a porosity rate of 71%, which was fabricated by using pure DMF as the solvent, displayed the smallest *k* of 1.51 at 10^5^ Hz. We also calculated the permittivity of the porous sPI films using two appropriate rules of mixtures (Maxwell–Garnett (MG) and Looyenga–Landau–Lifshitz (LLL)) [38,45]. These two models are suitable for a mixture that has spherical air inclusions (*k* = 1) in the polymer matrix. The predicted *k* values from the MG model were slightly lower, while the LLL model was more consistent with the measured data. Meanwhile, all of the porous sPI films exhibited extremely low dielectric loss with a loss tangent value of less than 0.005 at 10^5^ Hz. The low dielectric loss should also be ascribed to the introduction of air, which cannot induce an increase in the system’s polarization.

We also measured the electrical breakdown strength (*E*_b_) of the resultant porous sPI films. The measured data were analyzed using the Weibull distribution [46]. The Weibull cumulative distribution function is defined as

(1) PE=1−ⅇ−Eαβ

where *P*(*E*) is the cumulative failure probability; *E* represents the electric field strength at which the breakdown occurs; *α* is the scale parameter, which corresponds to an electric field strength when the cumulative failure probability of the sample reaches 63.2%; and *β* is the shape parameter of the Weibull distribution. The Weibull distribution function is usually rearranged by taking two logarithms:

(2) $$In\left(-\mathrm{In}\left(1-P\left(E\right)\right)\right)=\beta \mathrm{In}E-\beta \mathrm{In}\alpha$$

According to Equation (2), the slope β and the intercept −βIn⁡α can be easily extracted from a plot of $In\left(-\mathrm{In}\left(1-P\left(E\right)\right)\right)$ versus In⁡E. As shown in Figure 4d and Table S1 (in Supplementary Materials), the breakdown strength of sPI films decreased from 177.1 kV/mm to 30.3 kV/mm as the porosity increased from 0% to 71%. The significant deterioration in breakdown strength was mainly caused by the air voids embedded in the sPI matrix. It should be noted that the sPI film with a porosity rate of 48% could maintain a relatively high *E*_b_ of 102.7 kV/mm while giving a low-*k* value of 1.95.

The mechanical properties of sPI films are also crucial for their applications as insulating materials. Figure 4e displays the tensile strength and the elongation-at-break values of the sPI films with various porosities. As expected, the tensile strength of porous films underwent tremendous deterioration due to the introduction of porous structures. Additionally, the tensile strength of resulting films decreased with the increase in porosity. However, the elongation-at-break values of porous sPI films were comparable to that of the dense film. The sPI film with porosity of 48% even showed a larger elongation value at break, suggesting that this film is very promising as a low-*k* dielectric material for flexible electronics.

The thermal stability of the porous PI films was examined using TGA measurements. Figure 4f illustrates the TGA curves of several porous sPI films and the dense film. Despite their porosity, sPI films displayed similar thermal degradation characteristics, with the majority of decomposition happening between 500 and 600 °C in an inert nitrogen environment. T5% (the temperature at 5 wt% weight loss) and T10% (the temperature at 10 wt% weight loss) of the dense and porous PI films were nearly identical, indicating that porous PI films preserve the outstanding thermal stability of floriated sPI.

**Figure 5:**
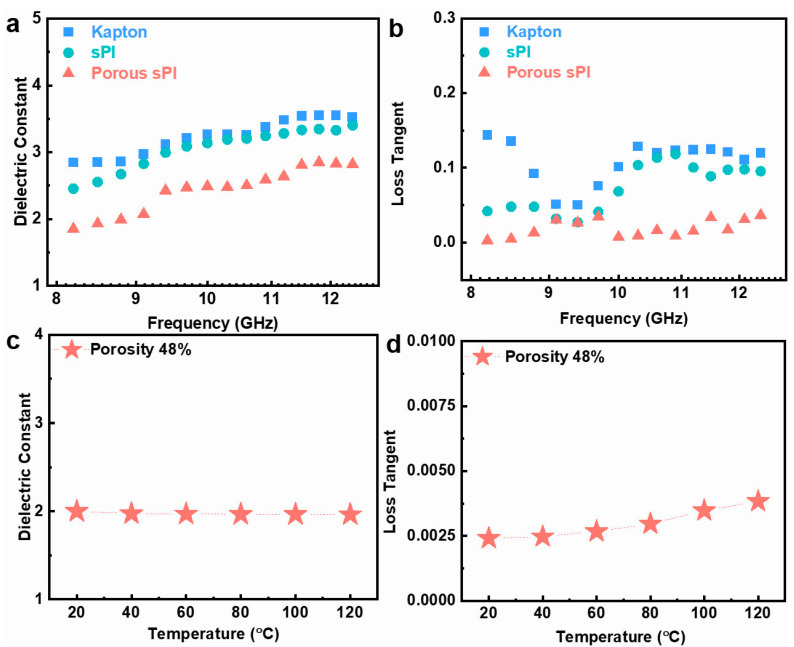
Dielectric properties of sPI films under GHz and various temperatures: (**a**,**b**) the dielectric constant and loss tangent of Kapton film, dense sPI film, and porous sPI film (with the porosity of 48%) in the frequency range of 8.2–12.5 GHz; (**c**,**d**) the variation in dielectric constant and loss tangent of the porous sPI film at 10^5^ Hz with the temperature.

For low-*k* dielectric materials, the dielectric performance in the UHF field is also an essential indicator. The dielectric characteristics of porous sPI films were investigated over an ultrahigh-frequency band of 8.2 to 12.5 GHz. Typical results are depicted in Figure 5a,b. Due to the presence of fluorinated groups, the dielectric constant and loss tangent of the dense sPI film were slightly lower than those of the Kapton film. In addition, the dielectric constant and loss tangent of sPI could be further reduced by generating a porous structure using the phase separation technique. These results are congruent with those found in the frequency range of 10^3^–10^7^ Hz discussed previously. We also studied the thermal stability of the porous sPI films’ dielectric properties. Taking the film with a porosity rate of 48% as an example, as depicted in Figure 5c,d, the *k* values and loss tangent were relatively stable between 20 and 120 °C. The practically unchanged *k* values indicate a weak temperature dependence of the dielectric constant. As the temperature increased, the loss tangent of the film increased slightly from 0.0025 to 0.0038. All of these findings suggest that the resulting porous sPI films are suitable for UHF microelectronics and 5G communication.

**Figure 6:**
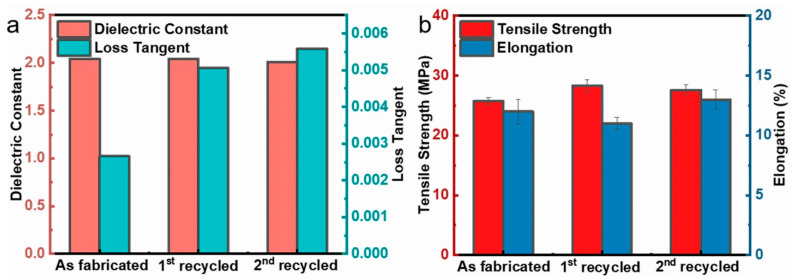
Recycle of porous sPI films: (**a**) dielectric constant and loss tangent at 10^5^ Hz; (**b**) tensile strength and elongation at break.

Traditional polyimide dielectrics are thermosetting and nonrecyclable. In contrast to the majority of previously reported polyimides, the sPI films synthesized in this paper are soluble. Therefore, it is possible to recycle the porous low-*k* dielectric films. We attempted to re-dissolve the sPI film with a 48% porosity in a DMF/CHCl_3_ mixed solvent (D/C = 6/4 in *wt*/*wt*) and re-prepare the porous film via the phase separation method. During the two dissolution and phase separation cycles, as depicted in Figure 6, the *k* values of the resulting porous sPI films were relatively stable (~2.0), with a slight increase in the dielectric loss from 0.002 to 0.005. Regarding mechanical properties, the tensile strength and elongation at break could be steadily maintained at 25 MPa and 12%, respectively. These findings demonstrate that porous sPI films can meet the requirements for the sustainable development of low-*k* dielectric materials and reduce material waste.

## 3. Experimental Section

### 3.1. Materials

N,N-Dimethylformamide (DMF, 99.8%), dimethylacetamide (DMAc, anhydrous, 99.8%), and trichloromethane (CHCl_3_, >98.0%) were purchased from Sigma-Aldrich. 4,4′-(Hexafluoroisopropylidene)diphthalic anhydride (6FDA, >98.0%), 2,2-bis[4-(4-aminophenoxy)phenyl]hexafluoropropane (BAPOFP, >98.0%) were obtained from Across. All the other chemicals used in the experiments were commercially available products. All chemicals were used as received without further purification. Kapton films with a thickness of 25 μm were purchased from DuPont and used as control samples.

### 3.2. Synthesis of Soluble Polyimide

The soluble polyimide (sPI) was synthesized from 6FDA and BAPOFP following a method previously described in the literature [43,44]. The details are as follows: To a completely dried 250 mL three-necked flask equipped with a mechanical stirrer, 6FDA (2.29 g, 5.15 mmol), BAPOFP (2.59 g, 5 mmol), and anhydrous DMAc (19.5 g) were added. Then, condensation polymerization was carried out at room temperature for 12 h under a nitrogen atmosphere and vigorous stirring. Next, a 1.5 mL mixture of acetic anhydride and pyridine with a volume ratio of 2:1 was added to the solution. After heating to 80 °C, the solution was further stirred for 3 h. Finally, the solution was slowly poured into an excessive amount of methanol/water (600/200 *v*/*v*) to obtain fibrous precipitates, which were filtrated, thoroughly washed with methanol/water, and dried under vacuum at 80 °C for 12 h to obtain sPI powders.

### 3.3. Preparation of Porous sPI Films

The porous sPI films were prepared via a nonsolvent-induced phase separation method. Firstly, the sPI powders were dissolved in various mixed solvents (DMF/CHCl_3_ = 10/0, 8/2, 6/4, and 4/6 in *wt*/*wt*) to obtain solutions with a polymer loading of 18.0 wt%. Secondly, specific volumes of the solutions were blade-coated on pre-cleaned glass plates with a blade gap of 150 µm. The wet films together with the glass plates were immediately transferred into a coagulation bath of water at 0 °C to form the porous structures. Finally, the porous sPI films were dried under vacuum at 80 °C for 2 h and then at 100 °C for another 10 h.

### 3.4. Characterizations

**Figure 7:**
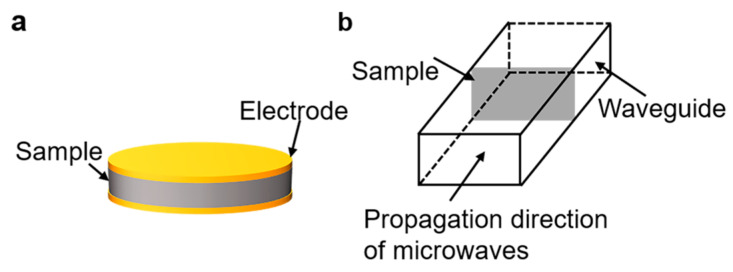
Schematic of (**a**) parallel plate method and (**b**) microwave method.

Nuclear magnetic resonance (NMR) spectra were recorded by using a Bruker DPX 400 spectrometer (400 MHz). Fourier transform infrared (FTIR) spectra were measured with a NICOLET 6700 spectrophotometer. The molecular weights of the sPI samples were tested using gel permeation chromatography (GPC, TRSEC MODEL 302) with DMF as the eluent. The column was calibrated using polystyrene standards. Scanning electron microscope (SEM) images were observed using SU8010 (Hitachi, Hitachi-shi, Japan). The samples were fractured in liquid nitrogen and sprayed with gold in advance. Mechanical tensile tests of the films were performed using a universal tester (ESM 303, Mark-10, New York, NY, USA). The sample size was 40 × 10 mm^2^, and the deformation rate in tensile tests was 13 mm/min. Five specimens were tested, and the averaged results were reported for each sample. The low-frequency dielectric properties of the films were measured using an impedance analyzer (E4900A, Keysight, Santa Rosa, CA, USA) in the frequency range of 20 to 3 × 10^7^ Hz and temperature range of 20–120 °C. The configuration is shown in Figure 7a. An AC voltage of 500 mV was applied, and a parallel coalescence of a capacitor and a resistor was used as the equivalent circuit model to simulate the behavior of the films. The complex impedance of the equivalent circuit can be denoted as follows:

(3) $$Ζ={Ζ}^{\prime }-j{Ζ}^{\prime \prime }=\frac{1}{j2\pi fC+\frac{1}{R}}$$

where *f* is the frequency, *C* is the capacitance, and *R* is the resistance. Thus, the dielectric constant (*k*) and loss tangent (*tan δ*) of the films can be calculated as follows:

(4) $$k=\frac{dC}{A\varepsilon_{0}}=\frac{dΖ\prime \prime }{j2\pi fC+\frac{1}{R}}$$

(5) $$tan\delta =\frac{Ζ\prime }{Ζ\prime \prime }$$

where *d* is the thickness and *A* is the surface area of the films. Gold electrodes with a diameter of 10 mm were sputtered on both surfaces of samples prior to tests. High-frequency dielectric properties at the X band (8.2–12.5 GHz) were characterized through the waveguide method using an Agilent E5071C S-parameter network analyzer. The Waveguide method, also known as the transmission/reflection method (TR), involves placing a sample in a section of a waveguide or coaxial line and measuring the two ports’ complex scattering parameters with a vector network analyzer. The waveguide used in this work is shown in Figure 7b. The measured sample size was 22.9 × 10.2 mm^2^. The electrical breakdown strength of the sPI films was measured using a high voltage source (KEITHLEY 2290-10, DC voltage, Beaverton, OR, USA) at room temperature, and the experimental data were analyzed with the two-parameter Weibull statistics. During the measurements, the increasing rate of voltage was set at 50 V/s. The thermal gravimetric analysis (TGA) of films was performed on TG/DTA 6300 (Hitachi, Japan) under an increasing rate of 10 °C·min^−1^ in nitrogen.

## 4. Conclusions

A series of porous low-*k* polyimide films were successfully fabricated through the nonsolvent-induced phase separation of a soluble fluorinated polyimide. By varying the formation of the binary mixed solvent, it was feasible to adjust the shape, size, and size distribution of pores formed in the sPI matrix. The porosity and average pore size fell from 71% to 33% and from 9.31 μm to 1.00 μm, respectively, when the mass ratio of DMF/CHCl_3_ increased from 10/0 to 4/6. The slower exchange rate between the mixed solvent and water with the increase in the CHCl_3_ content was found to be the primary cause of the evolving pore architecture. The permittivity and *E*_b_ of the resultant sPI films could be precisely controlled by adjusting the pore architectures. The porous films exhibited variable *k* values in the range of 1.51–2.42@100 kHz and *E*_b_ values between 30.3 and 119.7 kV/mm with the loss tangent less than 0.004@100 kHz. Meanwhile, the porous films illustrated excellent thermal stability, mechanical strength, and recyclable capability. All of these characteristics indicate that such porous sPI films are promising candidates for use in high-frequency communication devices as insulating dielectrics.

## Figures and Tables

**Table 1:** Porosity as well as mechanical and dielectric properties of the porous sPI films.

DMF/CHCl_3_, (*wt*/*wt*)	Thickness (μm)	Porosity (%)	Average Pore Size (μm)	Mechanical Properties		Dielectric Properties		
				Tensile Strength (MPa)	Elongation (%)	*k* @10^5^ Hz	*Tan δ* @10^5^ Hz	*E*_b_ (kV/mm)
10/0	57	71%	9.31	11.5	5.5	1.51	0.004	30.3
8/2	26	60%	2.47	19.1	7.1	1.73	0.003	40.4
6/4	20	48%	1.15	25.7	11.7	1.95	0.003	102.7
4/6	18	33%	1.00	37.8	7.3	2.42	0.003	119.7

**Table 2:** Hansen solubility parameters of all components in phase separation to fabricate porous films.

Components	MV (cm^3^/mol)	Solubility Parameter (MPa)^1/2^			Δδ_mix−water_
		δd	δp	δh	
sPI	517	21.7	7.0	5.7	
DMF	82.6	17.4	13.7	11.3	
CHCl_3_	80.7	17.8	3.10	5.70	
H_2_O	18.0	15.6	16.0	42.3	
DMF/CHCl_3_ 10/0		17.4	13.7	11.3	31.3
DMF/CHCl_3_ 8/2		17.5	12.3	10.6	32.1
DMF/CHCl_3_ 6/4		17.5	10.7	9.7	33.3
DMF/CHCl_3_ 4/6		17.6	8.7	8.6	34.7

## Data Availability

Not applicable.

## Supplementary Materials

The following supporting information can be downloaded at: https://www.mdpi.com/article/10.3390/molecules28073095/s1, Solubility parameters: Calculation of solubility parameters; Table S1: Fitting data and Weibull distribution parameters of sPI films.
