# Supplementary material for: Sustainable Dielectric Films with Ultralow Permittivity from Soluble Fluorinated Polyimide
Source: Molecules. 2023 Mar 30;28(7):3095. doi: 10.3390/molecules28073095 (PMC10096061; doi:10.3390/molecules28073095)
Supplement: Supplementary file 1 [file molecules-28-03095-s001.zip › molecules-2294932-supplementary.pdf]

Supplementary Materials

Sustainable Dielectric Films with Ultralow Permittivity from Soluble Fluorinated Polyimide

Hejian Li [1], Xiangyi Kong [1], Shixiao Wang [1], Min Gong [1,2], Xiang Lin [1,2], Liang Zhang [1,2], and Dongrui Wang [1,2,*]

1 Department of Chemistry and Chemical Engineering, School of Chemistry and Biological Engineering, University of Science and Technology Beijing, Beijing 100083, China
2 Beijing Key Laboratory for Bioengineering and Sensing Technology, University of Science and Technology Beijing, Beijing 100083, China
* Correspondence: wangdr@ustb.edu.cn

Solubility parameters: The Hansen solubility parameters (HSPs) are useful to determine the solvent strength and the solubility of polymers. δ_d, δ_p, and δ_h are HSPs attributed to dispersive forces, polar forces, and hydrogen bonding, respectively. The solubility parameter of the mixture (solvent/co-solvent mixture) is calculated using the following equation:

$$\delta_{i,j,s} = \frac{\Sigma_j (x_j v_j \delta_{i,j})}{\Sigma_j (x_j v_j)} \tag{S1}$$

where x is the molar fraction, v corresponds to the molar volume, and $\delta_{i,j}$ denotes the solubility parameter of a specific component j in the mixture.

The difference between the solubility parameters of two components can be expressed using the Hansen equation as follows:

$$\Delta\delta_{i-j} = \sqrt{4(\delta_{i,d} - \delta_{j,d})^2 + (\delta_{i,p} - \delta_{j,p})^2 + (\delta_{i,h} - \delta_{j,h})^2} \tag{S2}$$

Table S1. Fitting data and Weibull distribution parameters of sPI films.

Porous films (D/C ratio)	Linear fitting results			Weibull parameters	
	slope	ln(-ln(1-P(E))) intercept	R	β	α (kV/mm)
10/0	3.9	-13.3	0.95	3.9	30.3
8/2	4.4	-16.5	0.96	4.4	40.4
6/4	1.9	-8.8	0.95	1.9	102.7
4/6	5.1	-24.4	0.93	5.1	119.7
Dense sPI film	3.2	-16.0	0.95	3.2	177.1
